# Molecular Characterization of Twenty-Five Marine Cyanobacteria Isolated from Coastal Regions of Ireland

**DOI:** 10.3390/biology8030059

**Published:** 2019-08-07

**Authors:** Katie Shiels, Norma Browne, Fiona Donovan, Patrick Murray, Sushanta Kumar Saha

**Affiliations:** Shannon Applied Biotechnology Centre, Limerick Institute of Technology, Moylish Park, Limerick V94 E8YF, Ireland

**Keywords:** marine cyanobacteria, morphology, molecular characterization, 16S rRNA sequence, isoenzyme banding pattern, superoxide dismutase (SOD), malate dehydrogenase (MDH)

## Abstract

Twenty-five marine cyanobacteria isolated from Irish coasts were characterized based on their morphological characters and 16S rRNA gene sequence analysis. In addition, superoxide dismutase (SOD) and malate dehydrogenase (MDH) isoenzyme banding patterns were used to differentiate two morphologically ambiguous isolates. In this study, six new cyanobacteria-specific primers were designed, and a 16S rRNA gene of twenty-five morphologically diverse cyanobacteria was successfully PCR amplified (1198–1396 bps). Assembled 16S rRNA sequences were used both for a basic local alignment search tool (BLAST) analysis for genus-level identification and to generate a phylogenetic tree, which yielded two major clusters: One with morphologically homogenous cyanobacteria and the other with morphologically very diverse cyanobacteria. *Kamptonema okenii* and *Tychonema decoloratum* were isolated from a single field sample of Ballybunion and were originally identified as the same ‘*Oscillatoria* sp.’ based on preliminary morphological observations. However, an alignment of 16S rRNA gene sequences and SOD and MDH isoenzyme banding pattern analyses helped in differentiating the morphologically-indistinguishable ‘*Oscillatoria* sp.’. Finally, after a re-evaluation of their morphological characters using modern taxonomic publications, the originally identified ‘*Oscillatoria* sp.’ were re-identified as *Kamptonema okenii* and *Tychonema decoloratum*, thus supporting the polyphasic approach of cyanobacteria characterization.

## 1. Introduction

Cyanobacteria (AKA blue-green algae) are prokaryotic, oxygen evolving, photosynthetic, Gram-negative bacteria found in a wide variety of habitats including the Arctic, the Antarctic and hot springs. They can survive in extreme environmental conditions such as drought and desiccation, a range of salinity, nitrogen starvation, heat and cold shock, photooxidation, anaerobiosis, and osmotic and UV stress [1,2,3]. Morphologically, cyanobacteria are very diverse, including such forms as unicellular, trichomatous, filamentous, filaments with true or false type of branching, multicellular (with CO_2_ fixation in vegetative cells and N_2_ fixation in heterocysts), and perennation (akinetes and hormogones) [1].

Earlier, only botanists dealt with this group of organisms and called them algae. Later, Stanier et al. [4] placed them with bacteria and called them cyanobacteria because of their cellular structure, which is similar to that of Gram-negative bacteria. The major contribution to the taxonomy of this group is by botanists and was mainly based on morphological characters, but some of the morphological characters were found elastic or plastic under different environmental and culture conditions [5,6,7,8,9,10]. The unsatisfactory taxonomic status of this group of organisms has been known for many years, and the best example is the widely used laboratory strain *Anacystis nidulans*, which was assigned to four different genera by several authors within four years [11]. Cyanobacterial taxonomy, at present, uses criteria that include mostly molecular sequencing and genetic approaches. Several molecular biological methods have been applied for cyanobacterial taxonomy [12,13,14,15,16]. However, molecular sequencing methods are also not an absolute method, as, in many instances, they are not in good agreement with the morphology-based classification system. The genetic sequence analysis (e.g., *rpoC1*, PC-IGS and 16S rRNA) method has been growing gradually in conjunction with the predominantly accepted 16S rRNA gene sequence analysis method [17]. The most complete and most often used phylogenetic scheme of cyanobacteria that is presently available is based on 16S rRNA gene sequence analysis. Though some controversial taxonomic problems of this method have been solved, it cannot be used as an alternative and reliable method for cyanobacterial taxonomy up to the species level. To overcome this challenge, a polyphasic approach of cyanobacterial taxonomy is becoming popular.

Superoxide dismutases (SODs) are the major defense enzymes of aerobic organisms, including cyanobacteria, and are considered very stable because they remain active even when other proteins are modified under severe environmental conditions [18]. Based on the metal ions in their active sites, SODs may be of different types (e.g., Mn-SOD, Fe-SOD, Ni-SOD, Cu/Zn-SOD, Fe/Zn-SOD, Fe/Mn-SOD) [19,20,21]. In some cyanobacteria (*Anacystis nidulans*, *Anabaena variabilis* and *Plectonema boryanum*), Fe-SOD is localized in the cytosol, whereas Mn-SOD is found primarily in the membrane fraction [22]. Other cyanobacteria such as *Oscillatoria willei* BDU 130511 contain both Fe-SOD and Mn-SOD [20], and *Lyngbya arboricola* contain Mn-SOD, Fe-SOD and Fe/Mn-SOD [21]. Cyanobacteria have also been reported to contain Ni-SOD and Fe/Ni-SOD [23]. SOD isoenzyme banding patterns have previously been used for the taxonomic differentiation of haematophagous bugs *Rhodnius* spp. [24], woody plants such as *Larrea ameghinoi* and *Larrea nitida* [25], legume plants *Vigna unguiculata* [26], and *Lathyrus* spp. [27]. However, in regard to cyanobacterial taxonomy, there are no published data on superoxide dismutase except for an unpublished PhD thesis on marine cyanobacterial taxonomy and phylogeny that used four isoenzymes banding patterns (http://shodhganga.inflibnet.ac.in/handle/10603/116004).

Schenk et al. [28] studied the malate dehydrogenase (MDH) isoenzyme profile in eight cyanobacterial species with polyacrylamide disc gel electrophoresis and obtained a maximum of eight and a minimum of five bands with different relative mobilities (R_m_). They concluded that the malate dehydrogenase zymograms of cyanobacteria can be used as “fingerprints” and have been used successfully in some cases such as in *Anabaena* and *Nostoc* with shared bands; however, they were not so successful in determining the position of *Anacystis nidulans* within the order Chroococcales.

In this manuscript, we initiated the identification of twenty-five marine cyanobacteria based on morphological characterization and further molecular characterization based on 16S rRNA gene sequencing, basic local alignment search tool (BLAST) analysis, and phylogenetic analysis. Subsequently, we used superoxide dismutase (SOD) and malate dehydrogenase (MDH) isoenzyme banding patterns to resolve the ambiguity between two isolates using a polyphasic approach.

## 2. Materials and Methods

### 2.1. Cyanobacterial Isolates

A total of 25 cyanobacterial isolates were used, representing the morphological diversity of the present Biobank at Shannon Applied Biotechnology Centre (Shannon ABC), Limerick Institute of Technology. These isolates belong to 11 genera representing *Anabaena* (1 species), *Calothrix* (1 species), *Chlorogloea* (1 species, 2 isolates), *Hyella* (1 species), *Kamptonema* (1 species), *Leptolyngbya* (7 species, 8 isolates), *Phormidium* (3 species, 7 isolates), *Pseudanabaena* (1 species), *Schizothrix* (1 species), *Spirulina* (1 species) and *Tychonema* (1 species). The isolates were grown in an ASN-III (Artificial Seawater Nutrient) medium [29] in an environmental growth chamber (EGC M48, Environmental Growth Chambers, Chagrin Falls, OH, USA) under photosynthetically active radiation (PAR, 400–700 nm) of 45 µmol photons m^−2^ s^−1^ for a 16/8 h light/dark cycle at 20 ± 1 °C. All these pure cyanobacterial isolates were obtained through standard, at least 8–10 generations of purification steps, including spread plate, streak plate, and liquid culture. We repeated the process when necessary after microscopic observations. However, prior to DNA isolation, all cyanobacteria were further treated with the antibiotics ampicillin (50 µg·mL^−1^ final concentration) and tetracycline (25 µg·mL^−1^ final concentration) for 48 h, and they were then streaked on ASN-III agar plates to check their purity prior to DNA isolation.

### 2.2. Morphological Characterization and Identification

The identification and morphological characterization of the cyanobacteria were carried out using a compound light microscope (Nikon Alphaphot-2 YS2, Nikon, Kingston, UK) at 40X and 100X magnifications. To identify each isolate of the present study, all important key characters such as cell width, cell length, attenuation of apical cells, and sheath morphology were considered and compared to three main taxonomic publications for species identification [30,31,32].

### 2.3. DNA Isolation and PCR Amplification

Approximately 1 mg (fresh weight) of actively grown axenic cyanobacterial biomass was used to isolate the total DNA following the method of Saha et al. [33]. The PCR amplification of the 16S rRNA gene was carried out on an Applied Biosystems 2720 Thermocycler using cyanobacteria specific pair of primers (Table 1). We designed cyanobacterial 16S rRNA gene specific primers from a total of 37 sequences of 16S rRNA gene from the cyanobacterial complete genome database (Cyanobase, http://genome.microbedb.jp/cyanobase/) and randomly selected 20 partial 16S rRNA gene sequences obtained from NCBI Genbank (http://www.ncbi.nlm.nih.gov/genbank/) (unpublished thesis of Fiona Donovan [34]). PCR amplification was carried out using a total volume of 25 µL containing 1.8 mM MgCl_2_, 0.5 mM each of deoxynucleotide triphosphates (dATP, dTTP, dGTP and dCTP), 10 µM of each primer, 1U of OneTaq^®^ DNA Polymerase (New England Biolabs, Ipswich, MA, USA) and 1 µL of DNA (50–100 ng) template re-suspended in water. PCR conditions were: Initial denaturation at 96 °C for 4 min followed by 35 cycles of denaturation at 94 °C for 30 s, annealing at 50 °C for 30 s, elongation at 72 °C for 90 s, and a final elongation at 72 °C for 8 min. PCR products were resolved in 1% agarose gel, and the size of PCR bands were determined by comparing 1 kb ladder (New England Biolabs, Dublin, Ireland) run simultaneously. The gel image was captured by CCD camera and band analysis was carried out by Total Lab TL100 software (DNR Bio-imaging system, Neve Yamin, Israel).

### 2.4. DNA Sequencing, Editing and BLAST Analysis

PCR products were purified prior to sending for sequencing along with both forward and reverse primers. Briefly, PCR DNA in 200 µL of water was mixed with 200 µL of chloroform by gentle vortexing prior to centrifugation at 14,000× *g* for 3–4 min. The upper water layer containing DNA was transferred to a new tube, and 20 µL of 3M NaOAc (pH 5.2) was added. After gentle inversions, 200 µL of isopropanol was added, and the content was centrifuged at 14,000× *g* for 12 min. The DNA pellets thus obtained after decanting-off of supernatants were washed with 400 µL of 70% ethanol, spun briefly at 14,000× *g* for 2 min, allowed to air dry overnight, and finally dissolved in 40 µL of sterile distilled water. 5 µL of the DNA sample was used for quantification and quality check [33] prior to sending the samples at appropriate concentrations to LGC Genomics (Berlin, Germany) for Sanger Sequencing. The sequence information was obtained as AB1 type files that were analyzed using BioEdit 7.1.9 (Tom Hall, Carlsbad, CA, USA), where chimeras were resolved manually by comparing the peaks of the chromatogram with the corresponding base pair edits (addition, deletion and replacement). The analysis began by aligning the two sequences to find complementary regions using the online Clustal Omega multiple sequence alignment program (http://www.ebi.ac.uk/Tools/msa/clustalo/). Then, in the BioEdit platform, the sequence was edited by walking upstream and downstream of this complementary region, manually editing and/or deleting base pairs based on the careful analysis of the chromatogram peaks. Once the careful walking through the upstream region of the forward primer sequence and downstream region of the reverse primer sequence was completed, the total length sequence was obtained that contained the upstream region of the forward sequence, common regions of the two primers sequence, and the downstream region of the reverse primer sequence. The final complete sequence was then submitted to the BLAST (https://blast.ncbi.nlm.nih.gov/Blast.cgi?PAGE_TYPE=BlastSearch), for the possible identification of the cyanobacterial species/strain. All the edited sequences were then deposited with specific Genbank accession numbers using the online portal (https://submit.ncbi.nlm.nih.gov/).

### 2.5. Construction of Phylogenetic Tree

A phylogenetic tree based on 16S rRNA sequences of selected cyanobacteria was constructed using the “One Click” mode of the online software platform (http://www.phylogeny.fr/simple_phylogeny.cgi). The sequences were uploaded as FASTA format, and, upon submission, the default method started working to generate the tree. The “One Click” default method consists of four stages: (1) Multiple Alignment by MUSCLE (MUltiple Sequence Comparison by Log-Expectation), (2) curation via the Gblocks program that eliminates the poorly aligned positions and divergent regions, (3) phylogeny by PhyML, and (4) tree rendering by the TreeDyn tree drawing program. In this phylogenetic tree construction method, the usual bootstrapping procedure is replaced by a new confidence index (approximate likelihood-ratio test—aLRT) that is much faster to compute.

### 2.6. Preparation of Isoenzyme Samples and Native-PAGE

Samples for the activity staining of marker isoenzymes were prepared from thoroughly washed cyanobacterial cells re-washed with an extraction buffer (62.5 mM Tris-Cl, pH 6.8). Pellets were homogenized using a pre-chilled mortar and pestle in the presence of sterile sand powder, thus adding an ice-cold extraction buffer. The homogenized contents were centrifuged at 14,000× *g* for 15 min at 4 °C, and the supernatants were transferred to a new tube. Then, another round of centrifugation at above conditions were carried out to obtain clear supernatants, whose protein concentrations were estimated by Bradford reagent, B6916 (Sigma Chemicals, Arklow, Ireland) using Bovine Serum Albumin as standard [36]. Eight µg of total proteins and 16 µg of total proteins were loaded with native sample buffer that contained no denaturing agents for SOD and MDH, respectively. Then, electrophoresis was carried out using a Tris-glycine buffer (pH 8.3) in an ice-bath (~4 °C) with 1 mm thick polyacrylamide gels. Enzyme samples were resolved at 100 V for 40 min through the stacking gel (6%), followed by such at 120–140 V for 1 h through the separating gel (10%).

### 2.7. Native-PAGE Gel Staining for SOD (EC 1.15.1.1)

Activity staining for SOD on native-PAGE gel was carried out as described earlier [37]. Briefly, the gels were soaked in a staining solution (50 mL of 50 mM Tris-Cl (pH 8), 10 mg nitro blue tetrazolium (NBT) (VWR Chemicals, Radnor, PA, USA), 1 mg of ethylene-diamine-tetraacetic acid (EDTA) (Sigma Chemicals, Saint Louis, MO, USA) and 2 mg of riboflavin (Alfa Aesar, Ward Hill, MA, USA)) for 30 min in the dark at room temperature (~22 °C) and then illuminated with white fluorescent light (100 µmol photons m^−2^ s^−1^) until achromatic bands in the dark blue background were visible.

### 2.8. Native-PAGE Gel Staining for MDH (EC 1.1.1.37)

The activity staining of malate dehydrogenase (MDH, New Delhi, India) was carried out by immersing the gel in 50 mL of 50 mM Tris-Cl (pH 8.5) containing 150 mg of malic acid (Merck Millipore, Burlington, MA, USA), 10 mg of nicotinamide-adenine dinucleotide (NAD) (PanReac AppliChem, Darmstadt, Germany), 10 mg of NBT (VWR Chemicals), and 2 mg of phenazine methosulfate (PMS) (PanReac AppliChem). The gel was incubated at ~22 °C until the indigo bands appeared [38].

## 3. Results and Discussion

**Figure 1:**
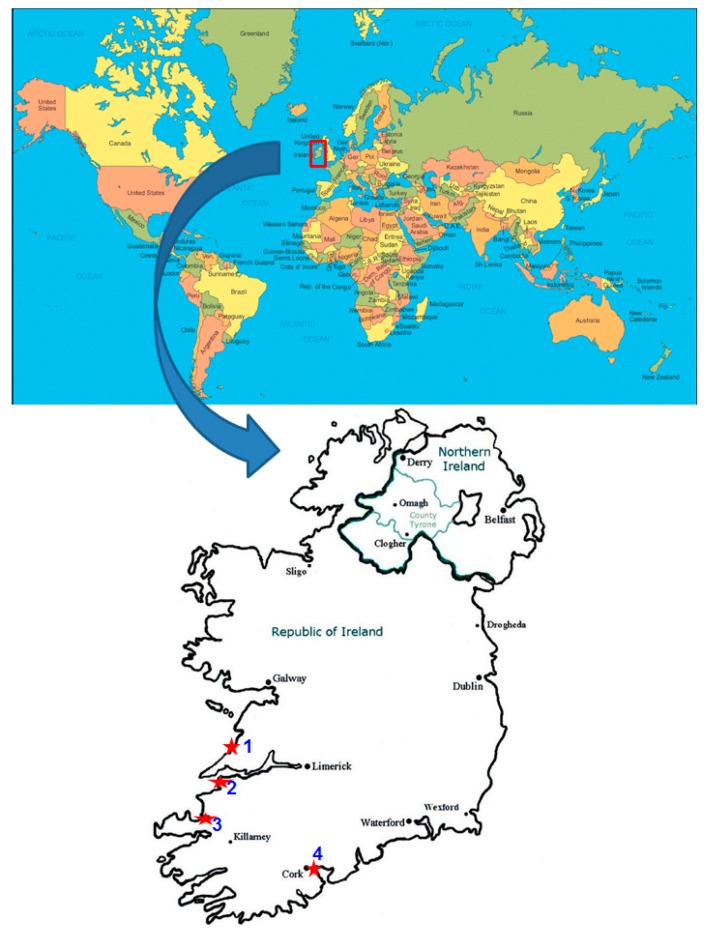
Map showing sampling sites of Irish coastal areas. 1, Kilkee; 2, Ballybunion; 3, Tralee; and 4, Cork.

**Figure 2:**
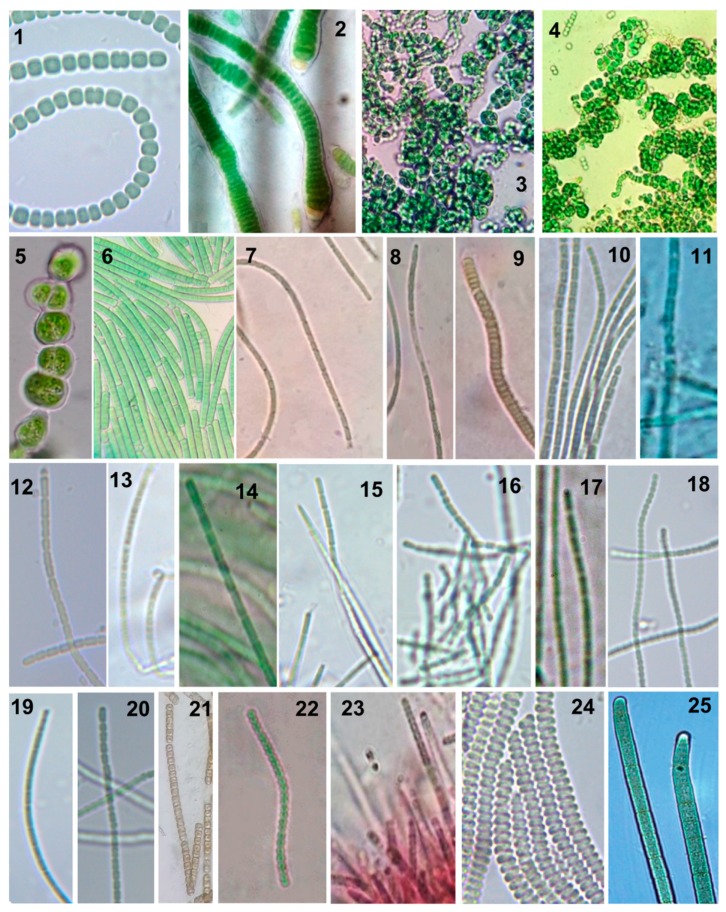
Photomicrographs showing morphological variations of marine cyanobacteria. 1, *Anabaena variabilis* SABC011501; 2, *Calothrix contarenii* SABC022701; 3, *Chlorogloea microcystoides* SABC011701; 4, *Chlorogloea microcystoides* SABC022904; 5, *Hyella gigas* SABC011201; 6, *Kamptonema okenii* SABC011902; 7, *Leptolyngbya africana* SABC021601; 8, *Leptolyngbya africana* SABC011801; 9, *Leptolyngbya ectocarpi* SABC012402; 10, *Leptolyngbya foveolarum* SABC011302; 11, *Leptolyngbya fragilis* SABC012503; 12, *Leptolyngbya fragilis* SABC031801; 13, *Leptolyngbya tenuis* SABC010201; 14, *Leptolyngbya valderiana* SABC022801; 15, *Phormidium angustissimum* SABC011301; 16, *Phormidium angustissimum* SABC020801; 17, *Phormidium angustissimum* SABC022612; 18, *Phormidium angustissimum* SABC022901; 19, *Phormidium angustissimum* SABC030403; 20, *Leptolyngbya norvegica* SABC031702; 21, *Phormidium* sp. SABC022903; 22, *Pseudanabaena minima* SABC031701; 23, *Schizothrix* sp. SABC022401; 24, *Spirulina subsalsa* SABC051501; and 25, *Tychonema decoloratum* SABC011901. Photomicrographs are not shown in scale (Refer to Table 3 for cell sizes).

Cyanobacterial isolates of the present manuscript represent the coastal sampling sites Ballybunion (11 isolates), Cork (1 isolate), Kilkee (9 isolates) and Tralee (4 isolates) of the Republic of Ireland (Table 2, Figure 1). Based on the micro-morphological characters observed under a light microscope, we identified a total of 19 species (morphological forms) of 11 genera *Anabaena*, *Calothrix*, *Chlorogloea*, *Hyella*, *Kamptonema*, *Leptolyngbya*, *Phormidium*, *Pseudanabaena*, *Schizothrix*, *Spirulina* and *Tychonema*, representing 25 cyanobacterial isolates of the present study. Table 3 and Figure 2 provide the detailed description of the morphological characters of all the isolates, which comprises only two heterocystous forms. The remaining are non-heterocystous trichomatous or filamentous forms. These isolates can be categorized into three cells-diameter range of 1–3 µm (17 isolates), 3–5 µm (five isolates), and 5–10 µm (three isolates) (Table 3). For molecular characterization, all cyanobacterial 16S rRNA genes were PCR amplified from total DNA using a newly designed cyanobacteria-specific pair of primers (Table 1). Successful PCR yielded PCR products of about 1198–1396 bps, as determined by agarose gel electrophoresis (Supplementary Figure S1) and edited sequences that were published at Genbank (Table 2). The size of PCR products obtained for 24 out of the 25 cyanobacteria tested suggest that the primers designed in this study are suitable for the amplification of above 1300 bps of the 16S rRNA gene of cyanobacteria (Table 2). Because of the availability of more cyanobacterial genome in the public database, it was possible to design new primers in this study that are capable of amplifying larger size 16S rRNA products, while starting cyanobacteria specific primers could only amplify approximately 700 bps [35].

**Figure 3:**
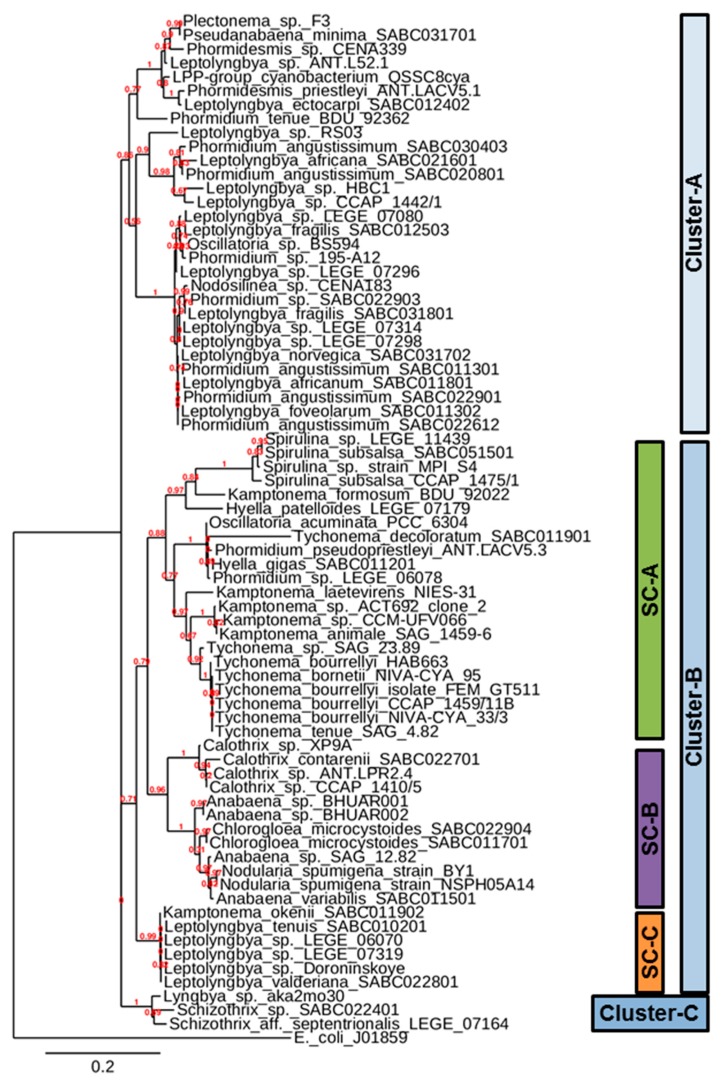
Phylogenetic tree of twenty-five cyanobacterial strains from the Shannon ABC biobank along with Genbank selected cyanobacteria based on their 16S rRNA sequences, outrooted with 16S rRNA sequence of *E. coli* (J01859). SC-A, sub-cluster-A; SC-B, sub-cluster-B and SC-C, sub-cluster-C.

Figure 3 represents the phylogenetic tree based on the 16S rRNA sequences of twenty-five marine cyanobacteria of the Shannon ABC biobank and 48 Genbank cyanobacterial 16S rRNA sequences. *E. coli* (J01859) 16S rRNA gene sequence from NCBI Genbank was used as out root organism for the construction of phylogenetic tree. The phylogenetic tree can broadly be divided into three clusters. Cluster-A can be considered as a homogenous cluster with almost similar morphotypes, consisting of *Phormidium*, *Plectonema*, *Pseudanabaena*, *Phormidesmis*, *Nodosilinea*, *Oscillatoria* and *Leptolyngbya*, all of which are filamentous cyanobacteria without heterocysts. The characteristics of the Irish isolates of Cluster-A include: A thallus that ismostly green to blue green in color, mucilaginous, long filaments with thin sheath, entangled trichomes, and apical cells usually flat rounded or conical rounded. Cells in this cluster are generally longer than they are broad except in *Leptolyngbya foveolarum,* whose cells are shorter than broad (Table 3).

Cluster-B is heterogeneous and consists of eleven different genera (*Anabaena*, *Calothrix*, *Chlorogloea*, *Hyella*, *Kamptonema*, *Leptolyngbya*, *Nodularia*, *Oscillatoria*, *Phormidium, Spirulina* and *Tychonema*) representing the orders Chroococcales, Oscillatoriales and Nostocales. *Hyella gigas* SABC011201 of the present study and *Hyella patelloides* LEGE 07179 of Portuguese Atlantic coast are members of Chroococcales, which remained slightly distantly distributed within the same sub-cluster sub-cluster-A (SC-A) of the phylogenetic tree. In a recent study, *Hyella patelloides* LEGE 07179 was proposed as a new species based on less than 97% similarity to the available 16S rRNA gene sequences in the databases [39]. The characteristics of *Hyella gigas* include pseudo-filaments bent or irregularly branched with cells in one or in many rows. Based on these morphological characteristics, *Hyella* spp. was considered an intermediate between unicellular and true filamentous forms [30], and this may be true from genetic relatedness, as reflected in sub-cluster SC-A of the phylogenetic tree (Figure 3).

The order Chroococcales is represented by unicellular organisms that have spherical, ovoid or cylindrical cells. The cells may aggregate in irregular colonies, being held together by the slimy matrix secreted during the colony growth. Both the two isolates of *Chlorogloea microcystoides* (Chroococcales), one from Ballybunion and another from Kilkee (Table 2), are distributed within the members of Nostocales species of the constructed phylogenetic tree. Sometimes the cells of *Chlorogloea microcystoides* form ‘Anabaena-like’ rows of barrel shaped cells with very irregularly shaped trichomes. Interestingly, both *Chlorogloea microcystoides* remain closely associated with *Anabaena variabilis* and other heterocystous cyanobacteria in the phylogenetic tree (Figure 3). *Anabaena variabilis* and *Calothrix contarenii* are the only Nostocales members of Irish isolates that remain closely associated with other heterocystous cyanobacteria in the phylogenetic tree (Figure 3). Both these species are considered multicellular and with heterocysts, the special cells for nitrogen fixation. However, *Anabaena variabilis* possess intercalary heterocysts and trichomes without sheaths, while *Calothrix contarenii* possess basal heterocysts and trichomes with sheaths (Table 3). Cluster-C is the smallest cluster, consisting of only two genera *Lyngbya* and *Schizothrix* of Oscillatoriales. Both of these genera have almost similar morphotypes such as trichomes with sheaths and non-heterocystous filamentous cyanobacteria.

The BLAST analysis of 16S rRNA gene sequences was done, as shown in Table 4 of each cyanobacteria identified most closely related cyanobacteria in Genbank. This helped mostly for genus-level identification or provided enough of a hint for the further identification of cyanobacteria based on modern morphology based system for cyanobacterial taxonomy. The 16S rRNA gene sequence of some cyanobacterial species possessed closest match in Genbank with multiple sequences of similar genus, such as for *Calothrix contarenii* SABC022701, *Leptolyngbya foveolarum* SABC 011302, *Leptolyngbya fragilis* SABC012503, *Leptolyngbya norvegica* SABC031702, *Leptolyngbya valderiana* SABC022801 and *Spirulina subsalsa* SABC051501 (Table 4). Some cyanobacterial 16S rRNA gene sequences showed a close match in Genbank, with only 1–2 sequence(s) of similar genera, such as for *Anabaena variabilis* SABC011501, *Chlorogloea microcystoides* SABC022904, *Leptolyngbya africana* SABC021601, *Leptolyngbya ectocarpi* SABC012402, *Phormidium angustissimum* SABC022901 and *Schizothrix* sp. SABC022401. 16S rRNA sequence of *Hyella gigas* SABC011201, *Pseudanabaena minima* SABC031701, *Kamptonema okenii* SABC011902 and *Tychonema decoloratum* SABC011901 showed closest match in Genbank with sequences of the genus from the LPP-group (*Lyngbya*, *Phormidium*, *Plectonema*) of cyanobacteria. Hence, the identification of these cyanobacteria were based only on morphological classification. An example is *Hyella gigas* SABC011201, which has the same distinct morphological differences as that of 16S rRNA-based closely associated members of the *Phormidium* genus. In fact, *Hyella* is considered as morphological transition between unicellular and filamentous cyanobacteria [30]. The alignment of only available 16S rRNA sequence of *Hyella patelloides* LEGE 07179 showed a high degree of similarity with the 16S rRNA sequence of *Hyella gigas* SABC011201 (Supplementary Figure S2).

*Kamptonema okenii* and *Tychonema decoloratum* were isolated from a single field sample of Ballybunion (Table 2) and were initially identified as same ‘*Oscillatoria* sp.’ based on morphological characterization. However, later, based on their culture behavior and growth rate (data not shown), it appeared that these are two different *Oscillatoria* species. The multiple alignment of the 16S rRNA gene sequences of *Kamptonema okenii* SABC011902 and *Tychonema decoloratum* SABC011901 were very dissimilar (Supplementary Figure S3). The BLAST analysis of 16S rRNA gene sequence of *Kamptonema okenii* SABC011902 with twenty-five available *Kamptonema* spp. in Genbank revealed a 91% similarity each. with *Kamptonema animale* SAG 1459-6, *Kamptonema* sp. CCM-UFV066 and *Kamptonema* sp. ACT692 clone two, covering 97% of query sequences (Table 4; data analyzed on 03/07/2019). The BLAST analysis of the 16S rRNA gene sequence of *Tychonema decoloratum* SABC011901 with the sixty-six available *Tychonema* spp. in Genbank revealed an 84% similarity each, with *Tychonema* sp. SAG 23.89 and *Tychonema bourrellyi* HAB663 covering 97–98% of query sequences and 90% sequence similarity with *Oscillatoria acuminata* strain PCC 6304 covering 98% of query sequence (Table 4; data analyzed on 03/07/2019). Both *Kamptonema okenii* SABC011902 and *Tychonema decoloratum* SABC011901 remained in the same Cluster-B but in different sub-clusters SC-C and SC-A, respectively, in the tree (Figure 3). *Kamptonema okenii* SABC011902 grouped with *Leptolyngbya* spp., represented a small sub-cluster SC-C and remained away from rest of the *Kamptonema* spp. sequences from Genbank. This possibly indicates the genotype of Irish *Kamptonema okenii* SABC011902 is much different and, hence, as sparsely distributed in the tree as that of *Kamptonema formosum* BDU 92022 in the sub-cluster SC-A (Figure 3). Interestingly, all Genbank *Tychonema* sequences remained closely associated with all Genbank *Kamptonema* sequences in the same sub-cluster SC-A, while the 16S rRNA sequence of *Tychonema decoloratum* SABC011901 remained slightly distantly in the same sub-cluster SC-A, thus indicating the genetic difference that *Tychonema decoloratum* SABC011901 can be considered a new variety. Earlier, it was found that the *Trichodesmium*-species from freshwater were molecularly different from marine types. The same study also suggested a need for the revision of the genus *Tychonema* as the sequences for *Tychonema tenue* placed it in a clade separate from the rest of the genus, forming a robust clade [40]. Recently, based on combined molecular, cytomorphological and ecological data, *Kamptonema* was derived as a new genus from the polyphyletic *Phormidium*, and it was based on a clonal population from thermal waters in Dax, France. This new genus *Kamptonema* corresponds to the type of species originally described as *Oscillatoria animalis* Agardh from thermal springs in Karlovy Vary, Czech Republic [41]. Therefore, the molecular identification of cyanobacteria and their phylogenetic positions are constantly evolving and could require a polyphasic approach, including multilocus phylogenies—especially for the above genera isolated from marine habitats [40]. Several cyanobacterial genera are still more or less limited, defined by both phylogenetic and morphological features, and it is likely that these identification ambiguities will be resolved precisely in the future upon generation of more genetic data particular to their habitats.

The SOD isoenzyme profiles of selected two cyanobacteria were essentially similar except for the three additional SOD bands (SOD7, SOD9 and SOD10) that were found in *Kamptonema okenii* SABC011902. Both *Tychonema decoloratum* SABC011901 and *Kamptonema okenii* SABC011902 possessed seven SOD (SOD1-6 and SOD8) bands in common (Supplementary Figure S4).

The malate dehydrogenase (MDH) isoenzyme profile of two selected cyanobacteria were slightly varied. *Tychonema decoloratum* SABC011901 possessed MDH5, while *Kamptonema okenii* SABC011902 possessed MDH4, MDH6, MDH8 and MDH9, which are not shared by these two cyanobacteria. Both *Tychonema decoloratum* SABC011901 and *Kamptonema okenii* SABC011902 possessed five MDH (MDH1-3, MDH7 and MDH10) bands in common (Supplementary Figure S4).

Initially, both *Kamptonema okenii* SABC011902 and *Tychonema decoloratum* SABC011901 were identified as ‘*Oscillatoria* sp.’ based on rapid morphological investigations. Later, based on multiple alignments of 16S rRNA gene sequences (Supplementary Figure S3) and two-marker enzymes (Supplementary Figure S4) characterizations, a revised detail and careful morphological characterization (Figure 2, Table 3) was undertaken. Finally, these two ‘*Oscillatoria* sp.’ were re-identified as *Kamptonema okenii* and *Tychonema decoloratum* using modern taxonomic literature. This indeed supports the importance of polyphasic cyanobacterial taxonomy as a solution for ambiguous cyanobacterial identification, as also suggested by other studies [1,17,40,42].

## 4. Conclusions

In the present manuscript, twenty-five marine cyanobacteria were characterized by conventional morphology-based and molecular 16S rRNA sequence-based methods. Additionally, a polyphasic approach was applied for the differentiation of morphologically ambiguous isolates as a recent method of cyanobacterial taxonomy. This study found that the superoxide dismutase (SOD) and malate dehydrogenase (MDH) isoenzyme banding patterns may also be suitable as molecular markers for cyanobacterial taxonomy.

## Figures and Tables

**Table 1:** Details of forward (1–4) and reverse (5–7) primers used in this study to amplify 16S rRNA gene of marine cyanobacteria.

No	Primer Name	Sequence (5′–3′)	Reference
1	CYA106F	CGGACGGGTGAGTAACGCGTGA	[35]
2	FDSKS_CyaF1	AGAGTTTGATCCTGGCTCAGGATG	Present study
3	FDSKS_CyaF2	TGCTTAACACATGCAAGTCGAACG	Present study
4	FDSKS_CyaF3	TAGTGGCGGACGGGTGAGTAAC	Present study
5	FDSKS_CyaR1	CACCTTCCGGTACGGCTACCTTG	Present study
6	FDSKS_CyaR2	TACAAGGCCCGGGAACGTATTCACC	Present study
7	FDSKS_CyaR3	GCATTGTAGTACGTGTGTAGCCCA	Present study

**Table 2:** List of Irish marine cyanobacterial strains of this study, their sampling sites, primer pairs used for amplification of 16S rRNA gene sequences, edited sequence size, and GenBank accession numbers.

	Species	Strain	Sampling Site	Primer Pairs	Edited Sequence Size (bp)	GenBank Accession Numbers
1	*Anabaena variabilis*	SABC011501	Ballybunion (Lat 52.511389, Lon 9.677496)	2, 5	1385	KX765290
2	*Calothrix contarenii*	SABC022701	Kilkee (Lat 52.685969, Lon 9.652605)	2, 5	1388	KT740998
3	*Chlorogloea microcystoides*	SABC011701	Ballybunion (Lat 52.511389, Lon 9.677496)	4, 5	1308	KY807916
4	*Chlorogloea microcystoides*	SABC022904	Kilkee (Lat 52.685969, Lon 9.652605)	3, 5	1345	KY807917
5	*Hyella gigas*	SABC011201	Ballybunion (Lat 52.511389, Lon 9.677496)	2, 5	1381	KX818207
6	*Kamptonema okenii*	SABC011902	Ballybunion (Lat 52.511389, Lon 9.677496)	2, 5	1369	KY807915
7	*Leptolyngbya africana*	SABC021601	Kilkee (Lat 52.685969, Lon 9.652605)	2, 5	1392	KT740999
8	*Leptolyngbya africana*	SABC011801	Ballybunion (Lat 52.511389, Lon 9.677496)	2, 5	1371	KX818206
9	*Leptolyngbya ectocarpi*	SABC012402	Ballybunion (Lat 52.511389, Lon 9.677496)	2, 5	1376	KX765291
10	*Leptolyngbya foveolarum*	SABC011302	Ballybunion (Lat 52.511389, Lon 9.677496)	2, 5	1384	KX818208
11	*Leptolyngbya fragilis*	SABC012503	Ballybunion (Lat 52.511389, Lon 9.677496)	2, 5	1377	KX818209
12	*Leptolyngbya fragilis*	SABC031801	Tralee (Lat 52.306668, Lon 9.857311)	3, 5	1352	KX818202
20	*Leptolyngbya norvegica*	SABC031702	Tralee (Lat 52.306668, Lon 9.857311)	2, 5	1385	KX818211
13	*Leptolyngbya tenuis*	SABC010201	Ballybunion (Lat 52.511389, Lon 9.677496)	3, 5	1335	KX765288
14	*Leptolyngbya valderiana*	SABC022801	Kilkee (Lat 52.685969, Lon 9.652605)	3, 5	1349	KY807918
15	*Phormidium angustissimum*	SABC011301	Ballybunion (Lat 52.511389, Lon 9.677496)	1, 5	1317	KX818204
16	*Phormidium angustissimum*	SABC020801	Kilkee (Lat 52.685969, Lon 9.652605)	2, 5	1382	KT740997
17	*Phormidium angustissimum*	SABC022612	Kilkee (Lat 52.685969, Lon 9.652605)	1, 5	1312	KX765287
18	*Phormidium angustissimum*	SABC022901	Kilkee (Lat 52.685969, Lon 9.652605)	2, 5	1396	KX818205
19	*Phormidium angustissimum*	SABC030403	Tralee (Lat 52.306668, Lon 9.857311)	1, 5	1317	KX818203
21	*Phormidium* sp.	SABC022903	Kilkee (Lat 52.685969, Lon 9.652605)	3, 5	1362	KT741000
22	*Pseudanabaena minima*	SABC031701	Tralee (Lat 52.306668, Lon 9.857311)	2, 5	1379	KX818210
23	*Schizothrix* sp.	SABC022401	Kilkee (Lat 52.685969, Lon 9.652605)	4, 6	1198	KX765289
24	*Spirulina subsalsa*	SABC051501	Cork (Lat 51.793171, Lon 8.268673)	3, 5	1313	KY807919
25	*Tychonema decoloratum*	SABC011901	Ballybunion (Lat 52.511389, Lon 9.677496)	3, 5	1343	KY807914

Note: Lat, latitude; lon, longitude.

**Table 3:** Specific morphological descriptions of cyanobacteria studied.

Forms	Description	Taxonomic Identification	Strains/Remarks
A	Thallus gelatinous, dark green, trichome without sheath, flexuous, heterocystous, cells barrel shaped to spherical, 3–5 µm broad, 5–6 µm long, heterocysts oval, 6 µm broad, sometimes up to 8 µm long, spores colorless or yellowish brown.	*Anabaena variabilis*	1
B	Thallus crustaceous, compact, firm, dull green, filaments densely arranged, filaments up to 1 mm long, trichome swollen at the base, 9–15 µm broad at base, sheath thick, colorless, trichome 6–8 µm broad in the middle, trichomes with basal, more or less spherical or hemispherical heterocysts, filament ending in a long hair.	*Calothrix contarenii*	1
C	Small but macroscopically visible thallus, wart-like, made up by union of a number of daughter dark-green colonies. Cells spherical, ellipsoidal or polygonal-rounded, 1–5 µm in diameter. Sometimes cells form Anabaena-like rows of barrel shaped cells with very irregularly shaped trichomes.	*Chlorogloea microcystoides*	2/Sheath of isolate SABC011701 is often yellowish brown.
D	Thallus microscopic to dot-like on agar plate, often gelatinous, pseudofilaments bent or irregularly branched with cells in one or in many rows forming a chroococcacean stage; pseudofilaments 100–125 µm long, base cells 5–7.5 µm broad, cells in pseudofilament up to 17 µm long, cells at tip of pseudofilament 5 µm long, mucilaginous sheath usually thin, cells content highly granular to homogenous, spherical to slightly angular sporangia found in proximal end producing endospores.	*Hyella gigas*	1
E	Thallus dark blue-green, trichomes mostly straight to slightly curved, trichomes 3.5–5 µm wide, cells1.5–4.5 µm long, end cell rounded and often bent without calyptra, cells shorter than broad, trichome attenuated, cell content partially minutely granular to homogenous, not distinctly constricted at cross walls.	*Kamptonema okenii*	1
F	Thallus green, mucilaginous, filaments long, mostly entangled, straight to irregularly curved, trichome 1–1.2 µm broad, 1.5–2 µm long, cells quadrate or slightly rectangular, indistinctly constricted, very thin sheath, apical cell conical round.	*Leptolyngbya africana*	2/SABC011801 filaments loosely entangled, cells 1–1.5 µm broad, 1.5–3–4 µm long, constrictions in old filaments are distinct, end cell round
G	Thallus yellowish brown to yellowish green, filaments densely entangled, curved or bent, fragile, hormogonia present (usually 2–5 cells long), sheath distinct in older cells, no evident sheath in hormogonia. Cells 1–1.2 µm broad, 1–1.5–2.5 µm long, apical cell rounded to conical rounded, without calyptra.	*Leptolyngbya ectocarpi*	1
H	Thallus mucilaginous, blue-green to dark green, filaments variously curved to parallelly arranged, sheath thin but firmly attached to the trichome, cells somewhat shorter than broad, 1–1.5 µm broad and 0.8–1.8 µm long, cross walls constricted in old filaments, cell content homogenous, apical cells flat rounded, no calyptra.	*Leptolyngbya foveolarum*	1
I	Thallus light blue green, mucilaginous, filaments entangled and variously curved, cells slightly longer than broad, sometimes quadrate, constricted at cross-walls, filaments easily broken apart, sheath diffluent, end cell conical, very long open-ended sheath,	*Leptolyngbya fragilis*	2/SABC012503 cells 1.5–2 µm broad, 2–3 µm long; SABC031801 cells 1.5 µm broad, 2–2.5–3 µm long.
J	Thallus brown or brownish green, some pseudobranching, sheaths initially thin or absent, cells ~1.5 µm broad, 1.5–2 (4) µm long, cells sometime quadrate, distinctly constricted at the cross walls, sheath later thick, older filament with sheath 2–3 µm, filaments often wavy within sheath, apical cell slightly elongated and tapered.	*Leptolyngbya norvegica*	1
K	Thallus membranous, irregularly expanded, very thin diffluent sheath, trichome olive-green or blue-green, straight or slightly bent but densely entangled, cells 1.2–1.5 μm broad, 1.5–2 μm long, cross wall constricted, cell content homogenous to slightly granular, end cells attenuated, mostly acute conical or round, no calyptra.	*Leptolyngbya tenuis*	1
L	Thallus mucilaginous, blue green to dark green, filaments long and strong, not easily breaking into short fragments, sheath thin, mucilaginous, cells longer than broad, 1.5 μm broad, 2.5–3 μm long, cross wall not distinctly constricted, granule on cross-wall, cell content homogenous to slightly granular, end cell acute conical to round.	*Leptolyngbya valderiana*	1
M	Thallus pale green to blue green, mucilaginous, filaments with thin but firm sheaths, cells longer than broad, trichomes bent and entangled densely or loosely, end cell mostly conical round, no calyptra, cross walls distinct in old filaments, cell content homogenous.	*Phormidium angustissimum*	5/SABC011301 cells 1 µm broad, 1.5 µm long; SABC020801 cells 0.8–1 µm broad, 1 µm long; SABC022612 cells 1–1.5 µm broad, 1.5–2 µm long; SABC022901 cells 1–1.2 µm broad, 1.5–2 µm long; SABC030403 filaments brownish, cells 0.8–1 µm broad, 1–1.5 µm long.
N	Thallus mucilaginous, distinct and firm sheath all through the trichome, cells longer than broad, 0.8–1 µm broad, 3–4 µm long, cross wall distinctly constricted, cell content homogenous, apical cell acute conical or round, no calyptra.	*Phormidium* sp.	1
O	Trichomes solitary or crowded in clusters, straight or almost straight, pale blue-green, cells barrel shaped, 1.2–1.5 µm broad, 1.2–1.5 µm long, intensely constricted at cross walls, no heterocysts or sheath, end cells round	*Pseudanabaena minima*	1
P	Thallus green in young stage and then red, mucilaginous, filaments densely entangled to radial fascicles, trichomes with fine sheath, sheath diffluent and colorless, pseudobranching present, trichomes 2 µm broad, cells 2–3 µm long, often quadratic, filaments long and randomly intertwined, cells constricted at cross walls but indistinct towards apices, end cells without calyptra, end cell rounded conical.	*Schizothrix* sp.	1
Q	Thallus mucilaginous, blackish green, trichomes solitary to entangled with other trichomes, regularly screw-like coils, 4.2–4.8 µm broad, 2.1–2.6 µm long, coils dextral, regularly tightly joined to one another, arranged nearly in parallel, rapidly motile with screw like rotation, end cells rounded.	*Spirulina subsalsa*	1
R	Thallus light blue-green, trichomes free-moving, mostly straight to slightly curved, cell content homogenous, cell constrictions not distinct, cells somewhat shorter than wide, 3.9–5.3 µm long, 6.5–8 µm wide, no heterocysts or sheath, end cells slightly bend round at one end, acute conical on the other end.	*Tychonema decoloratum*	1

**Table 4:** 16S rRNA gene sequences of marine cyanobacteria studied and their closest match in Genbank.

Morphotype	Fragment Size (bp)	Query Coverage (%)	Identity (%)	Closest Match in Genbank (Accession Number)
*Anabaena variabilis* SABC011501	1385	98	99	*Anabaena* sp. SAG 12.82 (KT290322.1)
		98	99	*Nodularia spumigena* strain BY1 (AF268004.1)
		99	98	*Nodularia spumigena* strain NSPH05A14 (AF268017.1)
*Calothrix contarenii* SABC022701	1388	98	98	*Calothrix* sp. CCAP 1410/5 (HF678513.1)
		98	97	*Calothrix* sp. ANT.LPR2.4 (AY493597.1)
		98	97	*Calothrix* sp. XP9A (AM230670.1)
*Chlorogloea microcystoides* SABC022904	1345	95	99	*Chlorogloea microcystoides* SABC011701 (KY807916.1)
		99	97	*Anabaena* sp. BHUAR002 (HM235817.1)
		98	97	*Anabaena* sp. BHUAR001 (HM235816.1)
*Hyella gigas* SABC011201	1381	94	91	*Hyella patelloides* LEGE 07179 (HQ832901.1)
		98	99	*Phormidium* sp. LEGE 06078 (HM217075.1)
		98	99	*Phormidium pseudopriestleyi* ANT.LACV5.3 (AY493600.1)
*Kamptonema okenii* SABC011902	1369	97	91	*Kamptonema animale* SAG 1459-6 (EF654087.1)
		97	91	*Kamptonema* sp. CCM-UFV066 (MG563377.1)
		97	91	*Kamptonema* sp. ACT692 clone 2 (MK247996.1)
*Leptolyngbya africana* SABC021601	1392	99	97	*Phormidium angustissimum* SABC020801 (KT740997.1)
		99	95	*Leptolyngbya* sp. CCAP 1442/1 (HE975019.1)
		99	95	*Leptolyngbya* sp. HBC1 (EU249120.1)
*Leptolyngbya ectocarpi* SABC012402	1376	99	98	*Phormidesmis priestleyi* ANT.LACV5.1 (AY493586.1)
		99	97	*Leptolyngbya* sp. ANT.L52.1 (AY493584.1)
		99	97	LPP-group cyanobacterium QSSC8cya (AF170758.1)
*Leptolyngbya foveolarum* SABC 011302	1384	100	99	*Leptolyngbya* sp. LEGE 07296 (HM217082.1)
		99	99	*Leptolyngbya* sp. LEGE 07298 (HM217044.1)
		99	99	*Leptolyngbya norvegica* SABC031702 (KX818211.1)
*Leptolyngbya fragilis* SABC012503	1377	99	99	*Leptolyngbya foveolarum* SABC011302 (KX818208.1)
		98	99	*Leptolyngbya* sp. LEGE 07080 (HM217085.1)
		99	99	*Leptolyngbya norvegica* SABC031702 (KX818211.1)
*Leptolyngbya norvegica* SABC031702	1385	99	99	*Leptolyngbya foveolarum* SABC011302 (KX818208.1)
		99	99	*Leptolyngbya* sp. LEGE 07314 (HM217061.1)
		99	99	*Leptolyngbya* sp. LEGE 07298 (HM217044.1)
*Leptolyngbya tenuis* SABC010201	1335	99	99	*Leptolyngbya valderiana* SABC022801 (KY807918.1)
		99	99	*Leptolyngbya* sp. LEGE 06070 (HM217074.1)
		98	93	*Phormidium tenue* BDU 92362 (KU958134.1)
*Leptolyngbya valderiana* SABC022801	1349	100	100	*Leptolyngbya* sp. LEGE 06070 (HM217074.1)
		100	99	*Leptolyngbya* sp. LEGE 07319 (HM217045.1)
		98	100	*Leptolyngbya* sp. Doroninskoye (KT753316.1)
*Phormidium angustissimum* SABC022901	1396	99	99	*Leptolyngbya norvegica* SABC031702 (KX818211.1)
		99	98	*Leptolyngbya* sp. LEGE 07298 (HM217044.1)
		99	97	*Phormidium* sp. 195-A12 (EU282429.1)
*Phormidium* sp. SABC022903	1362	98	99	*Leptolyngbya fragilis* SABC031801 (KX818202.1)
		98	99	*Nodosilinea* sp. CENA183 (KC695874.1)
		98	98	*Oscillatoria* sp. BS594 (KM019975.1)
*Pseudanabaena minima* SABC031701	1379	99	99	*Plectonema* sp. F3 (AF091110.1)
		99	97	*Leptolyngbya* sp. ANT.L52.1 (AY493584.1)
		99	96	Phormidesmis sp. CENA339 (KT731156.1)
*Schizothrix* sp. SABC022401	1198	99	96	*Schizothrix aff. septentrionalis* LEGE 07164 (KU951800.1)
		93	96	*Lyngbya* sp. aka2mo30 (AB863114.1)
		97	92	*Leptolyngbya* sp. RS03 (JF518829.1)
*Spirulina subsalsa* SABC051501	1313	99	99	*Spirulina* sp. LEGE 11,439 (KU951804.1)
		99	99	*Spirulina* sp. strain MPI S4 (Y18792.1)
		99	98	*Spirulina subsalsa* CCAP 1475/1 (HF678502.1)
*Tychonema decoloratum* SABC011901	1343	98	84	*Tychonema* sp. SAG 23.89 (KM019964.1)
		97	84	*Tychonema bourrellyi* HAB663 (FJ184385.1)
		98	90	*Oscillatoria acuminata* strain PCC 6304 (NR_102463.1)

## Supplementary Materials

The following are available online at https://www.mdpi.com/2079-7737/8/3/59/s1: Supplementary Figure S1, Representative 1% agarose gel showing PCR amplified products of 16S rRNA gene of selected cyanobacteria; Supplementary Figure S2, Alignment of 16S rRNA gene sequences of Hyella patelloides LEGE 07179 (Hyella_patelloides) and Hyella gigas SABC011201 (Hyella_gigas); Supplementary Figure S3, Alignment of 16S rRNA gene sequences of Tychonema decoloratum SABC011901 (TycDec) and Kamptonema okenii SABC011902 (KamOke); Supplementary Figure S4, Native-PAGE gels showing superoxide dismutase (SOD) and malate dehydrogenase (MDH) isoenzymes profile of Tychonema decoloratum SABC011901 and *Kamptonema okenii* SABC011902.

## References

[1] Pham H.T.L., Nguyen L.T.T., Duong T.A., Bui D.T.T., Doan Q.T., Nguyen H.T.T., Mundt S. (2017). Diversity and bioactivities of nostocacean cyanobacteria isolated from paddy soil in Vietnam. Syst. Appl. Microbiol..

[2] Sinha R.P., Häder D.-P. (1996). Photobiology and ecophysiology of rice field cyanobacteria. Photochem. Photobiol..

[3] Whitton B.A., Potts M. (2000). Introduction to the Cyanobacteria. The Ecology of Cyanobacteria, Their Diversity in Time and Space.

[4] Stanier R.Y., Sistrom W.R., Hansen T.A., Whitton B.A., Castenholz R.W., Pfennig N., Gorlenko V.N., Kondratieva E.N., Eimhjellen K.E., Whittenbury R. (1978). Proposal to place the nomenclature of the cyanobacteria (blue-green algae) under the rules of the International Code of Nomenclature of Bacteria. Int. J. Syst. Bacteriol..

[5] Anand N. (1988). Culture studies and taxonomy of blue-green algae-certain identification problems. Algol. Stud. Arch. Hydrobiol..

[6] Chang T.P. (1977). Sheath formation in *Oscillatoria aghardhii* Gomot. Schweiz. Z. Hydrobiology.

[7] Hoffmann L. (1985). Quelques remarques sur la classification des Oscillatoriaceae. Cryptogam. Algol..

[8] Hoffmann L., Demoulin V. (1985). Morphological variability of some species of Scytonemataceae (Cyanophyceae) under different culture conditions. Bull. K. Belg. Bot. Ver..

[9] Pearson J.E., Kingsbury J.M. (1966). Culturally induced variation in four morphologically diverse blue-green algae. Am. J. Bot..

[10] Wilmotte A. (1988). Growth and morphological variability of six strains of *Phormidium* cf. *ectocarpi* Gomont (Cyanophyceae) cultivated under different temperatures and light intensities. Algol. Stud. Arch. Hydrobiol..

[11] Komárek J. (1970). Genetic identity of the ‘*Anacystis nidulans’* strain Kratz-Allen/Bloom 625 with *Synechococcus* Näg. 1849. Arch. Protistenkd..

[12] Laamanen M.J., Forsström L., Sivonen K. (2002). Diversity of Aphanizomenon flos-aquae (Cyanobacterium) populations along a Baltic Sea salinity gradient. Appl. Environ. Microbiol..

[13] Laloui W., Palinska K.A., Rippka R., Partensky F., Tandeau de Marsac N., Herdman M., Iteman I. (2002). Genotyping of axenic and non-axenic isolates of the genus *Prochlorococcus* and the OMF-‘*Synechococcus*’ clade by size, sequence analysis or RFLP of the internal transcribed spacer of the ribosomal operon. Microbiology.

[14] Turner S., Huang T.-C., Chaw S.-M. (2001). Molecular phylogeny of nitrogen-fixing unicellular cyanobacteria. Bot. Bull. Acad. Sin..

[15] Wilmotte A., Bryant D.A. (1994). Molecular evolution and taxonomy of the cyanobacteria. The Molecular Biology of Cyanobacteria.

[16] Zheng W., Song T., Bao X., Bergman B., Rasmussen U. (2002). High cyanobacterial diversity in coralloid roots of cycads revealed by PCR fingerprinting. FEMS Microbiol. Ecol..

[17] Lee E., Ryan U.M., Monis P., McGregor G.B., Bath A., Gordon C., Paparini A. (2014). Polyphasic identification of cyanobacterial isolates from Australia. Water Res..

[18] Biemelt S., Keetman U., Mock H.-P., Grimm B. (2000). Expression and activity of isoenzymes of superoxide dismutase in wheat roots in response to hypoxia and anoxia. Plant Cell Environ..

[19] Kim E.J., Kim H.-P., Hah Y.C., Roe J.H. (1996). Differential expression of superoxide dismutase containing Ni and Fe/Zn in *Streptomyces coelicolor*. Eur. J. Biochem..

[20] Saha S.K., Uma L., Subramanian G. (2003). Nitrogen stress induced changes in the marine cyanobacterium *Oscillatoria willei* BDU 130511. FEMS Microbiol. Ecol..

[21] Tripathi S.N., Srivastava P. (2001). Presence of stable active oxygen scavenging enzymes superoxide dismutase, ascorbate peroxidase and catalase in a desiccation-tolerant cyanobacterium *Lyngbya arboricola* under dry state. Curr. Sci..

[22] Okada S., Kanematsu S., Asada K. (1979). Intracellular distribution of manganese and ferric superoxide dismutase in blue-green algae. FEBS Lett..

[23] Priya B., Premanandh J., Dhanalakshmi T.R., Uma L., Prabaharan D., Subramanian G. (2007). Comparative analysis of cyanobacterial superoxide dismutases to discriminate canonical forms. BMC Genom..

[24] Monteiro F.A., Lazoski C., Noireau F., Solé-Cava A.M. (2002). Allozyme relationships among ten species of *Rhodniini*, showing paraphyly of *Rhodnius* including *Psammolestes*. Med. Vet. Entomol..

[25] Lia V., Comas C.I., Cortés M.C., Hunziker J.H. (1999). Isozyme variation in *Larrea ameghinoi* and *Larrea nitida* (Zygophyllaceae): Genetic diversity and its bearing on their relationship. Genetica.

[26] Pasquet R.S. (1999). Genetic relationships among subspecies of *Vigna unguiculata* (L.) Walp. based on allozyme variation. Theor. Appl. Genet..

[27] Schifino-Wittmann M.T. (2001). Germplasm characterization of some *Lathyrus* species native to Rio Grande do Sul (Southern Brazil). Lathyrus Lathyrism Newsl..

[28] Schenk H.E.A., Hofer I., Metzner H. (1973). Malate-dehydrogenase isoenzyme enden als potentielles chemotaxonomisches kriterium für cyanophyceen-species. Biochem. Syst..

[29] Rippka R., Deruelles J., Waterbury J.B., Herdman M., Stanier R.Y. (1979). Generic assignments, strain histories and properties of pure cultures of cyanobacteria. J. Gen. Microbiol..

[30] Desikachary T.V. (1959). Cyanophyta.

[31] Komárek J., Anagnostidis K. (1998). Cyanoprokaryota, 1. Teil/1st Part: Chroococcales.

[32] Komárek J., Anagnostidis K. (2005). Cyanoprokaryota, 2. Teil/2nd Part: Oscillatoriales.

[33] Saha S.K., Uma L., Subramanian G. (2005). An improved method for marine cyanobacterial DNA isolation. World J. Microbiol. Biotechnol..

[34] Donovan F. (2014). Establishment of Irish Marine Cyanobacterial Germplasm and Characterisation of Selected Cyanobacteria for Industrially Potential Biomolecules. Master’s Thesis.

[35] Nübel U., Garcia-Pichel F., Muyzer G. (1997). PCR primers to amplify 16S rRNA genes from cyanobacteria. Appl. Environ. Microbiol..

[36] Bradford M.M. (1976). A rapid and sensitive method for the quantitation of microgram quantities of protein utilizing the principle of protein-dye binding. Anal. Biochem..

[37] Beauchamp C., Fridovich I. (1971). Superoxide dismutase: Improved assays and an assay applicable to acrylamide gels. Anal. Biochem..

[38] Wendel J.F., Weeden N.F., Soltis D.E., Soltis P.S. (1989). Visualization and interpretation of plant isozymes. Isozymes in Plant Biology.

[39] Brito Â., Ramos V., Mota R., Lima S., Santos A., Vieira J., Vieira C.P., Kaštovský J., Vasconcelos V.M., Tamagnini P. (2017). Description of new genera and species of marine cyanobacteria from the Portuguese Atlantic coast. Mol. Phylogenet. Evol..

[40] Komárek J., Kaštovský J., Mareš J., Johansen J.R. (2014). Taxonomic classification of cyanoprokaryotes (cyanobacterial genera) 2014, using a polyphasic approach. Preslia.

[41] Strunecky O., Komarek J., Šmarda J. (2014). *Kamptonema* (Microcoleaceae, Cyanobacteria), a new genus derived from the polyphyletic *Phormidium* on the basis of combined molecular and cytomorphological markers. Preslia.

[42] Bravakos P., Kotoulas G., Skaraki K., Pantazidou A., Economou-Amilli A. (2016). A polyphasic taxonomic approach in isolated strains of cyanobacteria from thermal springs of Greece. Mol. Phylogenet. Evol..

