# Supplementary material for: Molecular Characterization of Twenty-Five Marine Cyanobacteria Isolated from Coastal Regions of Ireland
Source: Biology (Basel). 2019 Aug 7;8(3):59. doi: 10.3390/biology8030059 (PMC6784279; doi:10.3390/biology8030059)
Supplement: Supplementary file 1 [file biology-08-00059-s001.pdf]

biology

Molecular Characterization of Twenty-Five Marine Cyanobacteria Isolated from Coastal Regions of Ireland

Supplementary Material

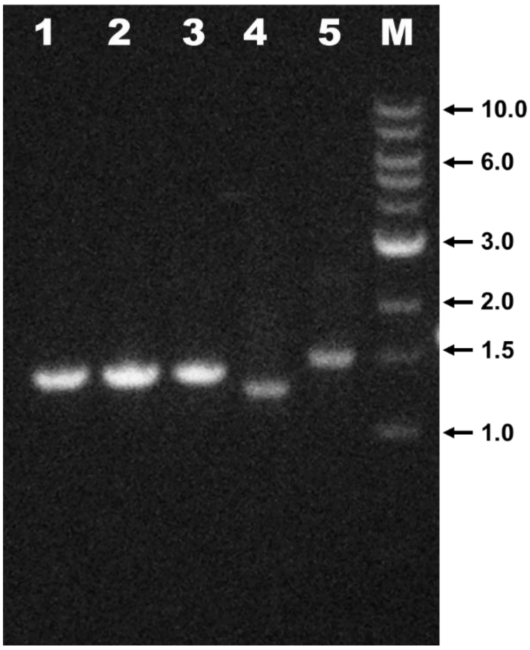

Figure S1. Representative 1% agarose gel showing PCR amplified products of 16S rRNA gene of selected cyanobacteria. Lane 1, *Chlorogloea microcystoides* SABC011701; Lane 2, *Chlorogloea microcystoides* SABC022904; Lane 3, *Kamptonema okenii* SABC011902; Lane 4, *Tychonema decoloratum* SABC011901; Lane 5, *Leptolyngbya valderiana* SABC022801; M, 1 kb DNA ladder (New England Biolabs).

```
CLUSTAL O(1.2.4) multiple sequence alignment


Hyella_patelloides      ctcagaatgaacgctggcggtatgcttaacacatgcaagtcgaacgaattcttcggaatg    60
Hyella_gigas            -----------------cagtaccg--tgcag---tcgacggtgaaccctcgggttcata    38
                                         * ***       **          *       **      **

Hyella_patelloides      agtggcggacgggtgagtaacg-cgtgagaacctgccttcaggatggggacaacagttgg    119
Hyella_gigas            gatggcggacgggtgagtaacacgtgtagaatctgcctccaggtcggggacaacagcggg    98
                          *******************      **** ****** ****  ***********  **

Hyella_patelloides      aaacgactgctaatacccaataagccgcaaggtaaaagatttatcgcctgaagaggggct    179
Hyella_gigas            aaactgctgctaatacccgatgtgcctaagggtgaaagattaattgcctggagatgagct    158
                        ****  ************ **  ***  * *** ******* ** ***** *** * ***

Hyella_patelloides      cgcgtccgattagctagttggtgaggtaaaggcttaccaaggcagcgatcggtagctggt    239
Hyella_gigas            cgcgtccgattagctagttggtagagtaaaagcctaccaaggctccgatcggtagctggt    218
                        **********************   ***** ** *********  ***************

Hyella_patelloides      ctgagaggacgagcagccacactgggactgagacacggcccagactcctacgggaggcag    299
Hyella_gigas            ctgagaggatgagcagccacactgggactgagacacggcccagactcctacgggaggcag    278
                        ********* **************************************************

Hyella_patelloides      cagtggggaattttccgcaatgggcgcaagcctgacggagcaataccgcgtgagggagga    359
Hyella_gigas            cagtggggaattttccgcaatgggcgaaagcctgacggagcaagaccgcgtgggggatga    338
                        ************************** **************** ******** **** **

Hyella_patelloides      aggcttttgggttgtaaacctcttttctcaaggaagaagacctgacggtacttgaggaat    419
Hyella_gigas            aggctcttgggttgtaaaccccttttctcagggaagaataactgacggtacctgaggaat    398
                        ***** ************** ********* ******* * ********** ********

Hyella_patelloides      cagcatcggctaactccgtgccagcagccgcggtaatacggaggatgcaagcgttattcg    479
Hyella_gigas            aagcatcggctaactccgtgccagcagccgcggtaagacggaggatgcaagcgttatccg    458
                         *********************************** ******************** **

Hyella_patelloides      gaattattgggcgtaaagcgtccgcaggtggtgcctcaagtctgctgtcaaagaccgaag    539
Hyella_gigas            gaattattgggcgtaaagcgttcgtaggtggctgttcaagtctgccgttaaagaccgggg    518
                        ********************* ** ******    ********** ** ********  *

Hyella_patelloides      cttaacttcgggcaggcagtggaaactgaagcactagagtacggtaggggtagagggaat    599
Hyella_gigas            cttaactccggaaaaactgtggaaactgaacagctagagtatggtaggggtaaggggaat    578
                        ******* ***  *  * ************   ******** **********  ******

Hyella_patelloides      ttccagtgtagcggtgaaatgcgtagagattggaaagaacaccagtggcgaaggcgctct    659
Hyella_gigas            tcccggtgtagcggtgaaatgcgtagatatcgggaagaacaccggtggcgaaggcgcctt    638
                        * ** ********************** ** ** ********* *************  *

Hyella_patelloides      actggaccgtaactgacactcagggacgaaagctagggtagcgaaagggattagataccc    719
Hyella_gigas            actgggccataactgacactgagggacgaaagctaggggagcgaaagggattagataccc    698
                        ***** ** *********** ***************** *********************

Hyella_patelloides      ctgtagtcttagctgtaaacgatggatactaggcgttgcttgtatcgacccgagcagtgc    779
Hyella_gigas            ctgtagtcctagctgtaaacgatggatactaggtgttgcccgtatcgacccgggcagtgc    758
                        ******** ************************ *****  *********** *******

Hyella_patelloides      cgtagccaacgcgttaagtatcccgcctggggagtacgcacgcaagtgtgaaactcaaag    839
Hyella_gigas            cgtagctaacgcgttaagtatcccgcctggggagtacgcacgcaagtgtgaaactcaaag    818
                        ****** *****************************************************

Hyella_patelloides      gaattgacgggggcccgcacaagcggtggagtatgtggtttaattcgatgcaacgcgaag    899
Hyella_gigas            gaattgacgggggcccgcacaagcggtggagtatgtggtttaattcgatgcaacgcgaag    878
                        ************************************************************

Hyella_patelloides      aaccttaccagggcttgacatcccacgaattctggggaaactcagaagtgccttcgggaa    959
Hyella_gigas            aaccttaccagggcttgacatgtccagaatcttggggaaacctaagagtgccttcgggag    938
                        *********************  *  ****  *********  *  *************

Hyella_patelloides      cgtggagacaggtggtgcatggctgtcgtcagctcgtgtcgtgagatgttgggttaagtc    1019
Hyella_gigas            ctggaacacaggtggtgcatggctgtcgtcagctcgtgtcgtgagatgttgggttaagtc    998
                        *  * * *****************************************************

Hyella_patelloides      ccgcaacgagcgcaaccctcgtccttagttgccatcattaagttgggcactttagggaga    1079
Hyella_gigas            ccgcaacgagcgcaaccctcgtccttagttgccatcattaagttgggcactttagggaga    1058
                        ************************************************************

Hyella_patelloides      ctgccggtgacaaaccggaggaaggtggggatgacgtcaagtcagcatgccccttacgct    1139
Hyella_gigas            ctgccggtgacaaaccggaggaaggtggggatgacgtcaagtcagcatgccccttacgtc    1118
                        **********************************************************

Hyella_patelloides      ctgggctacacacgtactacaatggtcaggacaaagggcagccaactcgcgagagtgagc    1199
Hyella_gigas            ctgggctacacacgtactacaatgggaaggacagagggtagcaagcgcgcgagtgcaagc    1178
                        *************************  ****** **** *** * * ****** *  ***

Hyella_patelloides      gaatctcatcaaacctggccacagttcagattgcaggctgcaactcgcctgcatgaagga    1259
Hyella_gigas            caatcccat-aaacctttcctcagttcagattgcaggctgcaactcgcctgcatgaaggc    1237
                         **** *** ******  ** **************************************

Hyella_patelloides      ggaatcgctagtaatcgcaggtcagcatactgcggtgaatccgttcccgggccttgtaca    1319
Hyella_gigas            ggaatcgctagtaatcgcaggtcagcatactgcggtgaatacgttcccgggccttgtaca    1297
                        **************************************** *******************

Hyella_patelloides      caccgcccgtcacaccatggaagctggccac-gcccgaagtcgttaccctaacccttttt    1378
Hyella_gigas            caccgcccgtcacaccatggcaagttggccatgcccgaagtcattaccctaaccgcaagg    1357
                        ******************** *  * * *   ********** ***********
```

Figure S2. Alignment of 16S rRNA gene sequences of *Hyella patelloides* LEGE 07179 (Hyella_patelloides) and *Hyella gigas* SABC011201 (Hyella_gigas).

```
CLUSTAL O(1.2.4) multiple sequence alignment

TycDec          ------------------cacttcggt---ctgtggcggatgagtgagta-cacatga-g
KamOke          acagtgcagtcgacggagtacttcggtacttagtggcggacgggtgagtaacgcgtgaga
                                   ********    :******** *.******* *.*.*** .

TycDec          atctgcctccaggtcagggacaacagcgggaaactgctgctaataccctatgtgcctaag
KamOke          atctgcccttaggagggggacaacagttggaaacgactgctaataccccatatgccgaga
                *******   ***: .**********  ****** .************ **.**** *..

TycDec          ggtgaatgattaattgcctggtgatcagctcgcgtccgatcagcttgttggtatagtaaa
KamOke          ggtgaaacattaatggcctgaggaggagctcgcgtctgattagctagttggtggggtaaa
                ******: ****** *****. **  ********** *** ****:******. .*****

TycDec          agcctacc-gggctccgatcggtagctggtctgagatgatgagcagccacgctggtacta
KamOke          ggcctaccaaggcgacgatcagtagctggtctgagaggatgatcagccacactgggactg
                .******* .*** .*****.*************** ***** *******.**** ***.

TycDec          aaacacggcccctactcctacaggaggcagcagtgaggaattttccgcggtgggcgaaat
KamOke          agacacggcccagactcctacgggaggcagcagtggggaattttccgcaatgggcgaaag
                *.*********. ********.*************.************..*********

TycDec          cctgacggagcgcgaccgcgtgggggatgaacgctctggtgttgtaaactccttttctca
KamOke          cctgacggagcaagaccgcgtgagggaggaaggtctgtggattgtaaacctcttttctca
                ***********..*********.**** *** *     * .********  *********

TycDec          gggaagaattactgacggtacctgaagaataatcatcggctaactccgtgccagcagccg
KamOke          gggaagaagaactgacggtacctgaggaatcagcatcggctaactccgtgccagcagccg
                ******** :***************.****.* ***************************

TycDec          cgatacgacggaggatgcaagcgttatccggtattattgggcgtaaagcgttcgtaggtg
KamOke          cggtaatacggaggatgcaagcgttatccggaattattgggcgtaaagcgtccgtaggtg
                **.**. ************************:******************* ********

TycDec          gctgttctagtctgccgttaaagaccggtccttaactcctgaacaactgtgaaaactgaa
KamOke          gctaagcaagtctgctgttaaagagcggagctaaactccgtaaaggcggtggaaactgca
                ***.: *:******* ******** ***: **:******  **...* ***.******.*

TycDec          cagctatattatggtaggagtaaggagaattcctggtgtagcgatgaaatgcgtagatat
KamOke          tagctagagtgtggtaggggtagagggaattcccagtgtagcggtgaaatgcgtagatat
                 ***** * *.*******.***..*.******* .********.****************

TycDec          cgggaagaacaccggtggcgaaggcgccttactgggccataactgacactgaacgacgac
KamOke          tgggaagaacaccggtggcgaaggcgctctactgggccacaactgacactgatggacgaa
                 **************************  ********** ************: *****.

TycDec          tgctagggcagcgaaagggattagatactcctgtagtcctagctgtcaacgatggatact
KamOke          agctaggggagcgaaagggattagataccccctgtagtcctagccgtaaacgatgaacact
                :******* ******************* ************** **.*******.* ***

TycDec          aggtgttgctcgtatcgactcgagcagtgccgtagctaacgcgttaagtatcccgcctgg
KamOke          aggtgttgcccgtatcgacccgggcagtgccgtagccaacgcgttaagtgttccgcctgg
                ********* ********* **.************* ************.* ********

TycDec          gtagtacgcacgctattgtgatactcagaggtgttgacgggggcccgcacaagcggtgga
KamOke          ggagtacgctcgcaagagtgaaactcaaaggaattgacgggggcccgcacaagcggtgga
                * *******:***:* :****:*****.***:.***************************

TycDec          gtatgttgttttattcgatgcgacgcgaagaaccttacgagggcttgacttgtccagaat
KamOke          gtatgtggtttaattcgatgcaacgcgaagaaccttaccaaggcttgacatgccgcgaat
                ****** ****:*********.**************** *.********:** * .****

TycDec          cttgaggaaacctaatactgccttcaggagctggaacacatgtggtgcatggctgtcgtc
KamOke          ccttcagagatgagggagtgcctacgggagcgcggacacaggtggtgcatggctgtcgtc
                * *  ..**.*   :.. * *****:*.*****  *.***** ******************

TycDec          agctcgtgtcatgagattttgggttaagtcgcgcaaagagcgcaactctcgtccttagtc
KamOke          agctcgtgtcgtgagatgttgggttaagtcccgcaacgagcgcaaccctcgttcttagtt
                **********.****** ************ *****.********* ***** ******

TycDec          gctatcattaagttgggcactttagggagactgcgggagacataccggaggaaggtgggg
KamOke          gccatcattaagttgggcactctagggagactgccggtgacaaaccggaggaaggtgagg
                ** ****************** ************ **:****:***************.**

TycDec          atgatgtcatgttagtatgccctatatgtcctgggctacacatgtactacaatgggaagg
KamOke          atgacgtcaagtcatcatgccccttacgtcttgggctacacacgtactacaatgctgcgg
                **** ****:** *  ****** :** *** *********** ***********  ..**

TycDec          acagagggtagcaagcgcgcgagtgcaagcctatcctataaaccttttctcagttcagat
KamOke          acaaagggcagccaaccagcgatggtgagcaaatcccataaaccgtggctcagttcagat
                ***.**** ***.*.* .****  * .***.:**** ******* *  ************

TycDec          cgcagggtgcatctcgcgtgcgtgaagggggtatcgcttgttatcgcatgtcagcatagt
KamOke          tgcaggctgcaactcgcctgcatgaaggaggaatcgctagtaatcccaggtcagcatact
                 ***** ****:***** ***.******.**:******:**:*** ** ********* *

TycDec          gcggtgaatgcgttcccgggccttgtacacaccgcctgtcacaccatggaagttggacat
KamOke          ggggtgaatacgttcccgggccttgtacacaccgcccgtcacaccatgggagttggccac
                * *******.*************************** ************.******.**

TycDec          gccagatgtcattactctc--agtgcaaggagggggatgccgatgcatt
KamOke          gcccgaagtcgttactccaaccgttcgcggaggaggacgcctgaatggt
                ***.**:***.******  .  .** *..*****.*** ***  .:.  . *
```

Figure S3. Alignment of 16S rRNA gene sequences of *Tychonema decoloratum* SABC011901 (TycDec) and *Kamptonema okenii* SABC011902 (KamOke).

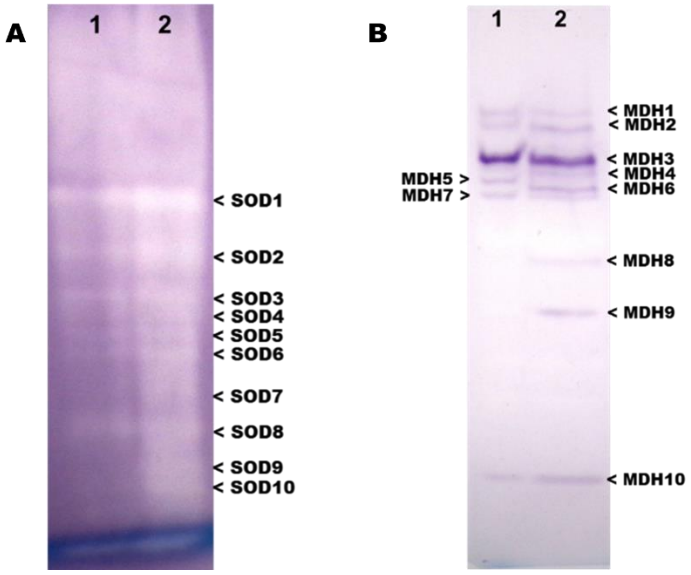

Figure S4. Native-PAGE gels showing superoxide dismutase (SOD) (A) and malate dehydrogenase (MDH) (B) isoenzymes profile of *Tychonema decoloratum* SABC011901 (lane 1) and *Kamptonema okenii* SABC011902 (lane 2).
